# A Rare Knee Fracture with Underestimated Severity

**DOI:** 10.5811/cpcem.2018.7.38817

**Published:** 2018-08-15

**Authors:** Shinsuke Takeda, Katsuyuki Iwatsuki, Akihiko Tabuchi, Sadahiro Kubo, Satoshi Teranishi, Hitoshi Hirata

**Affiliations:** * Anjo Kosei Hospital, Emergency and Critical Care Center, Anjo, Japan; † Nagoya University Graduate School of Medicine, Department of Hand Surgery, Nagoya, Japan

## CASE PRESENTATION

**Image 1:**
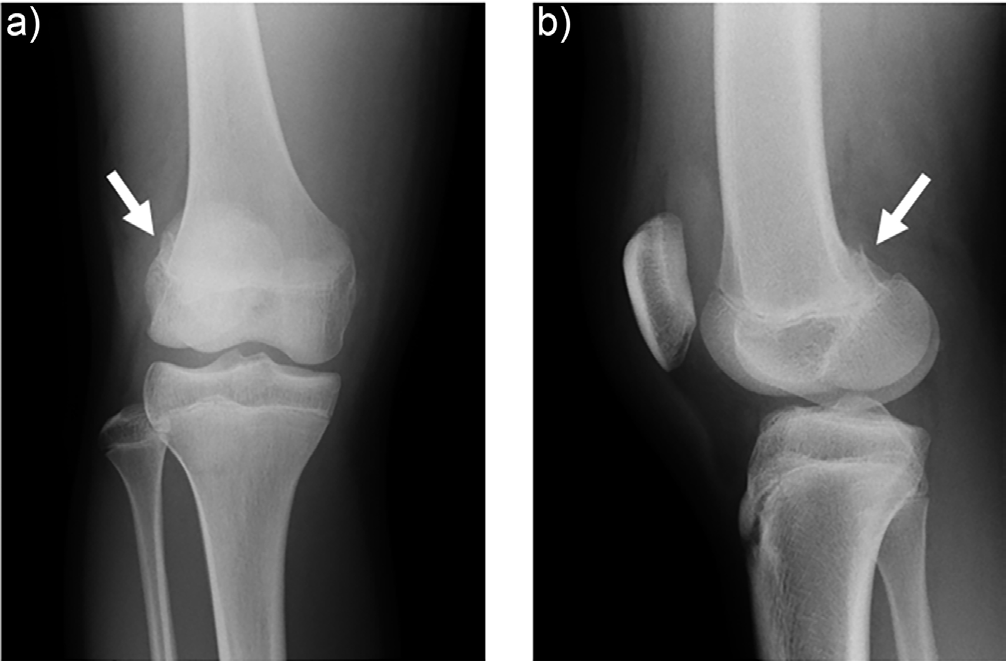
Anteroposterior a) and lateral b) radiograph show the injured knee with a minimal fracture of the lateral femoral condyle (arrow).

**Image 2:**
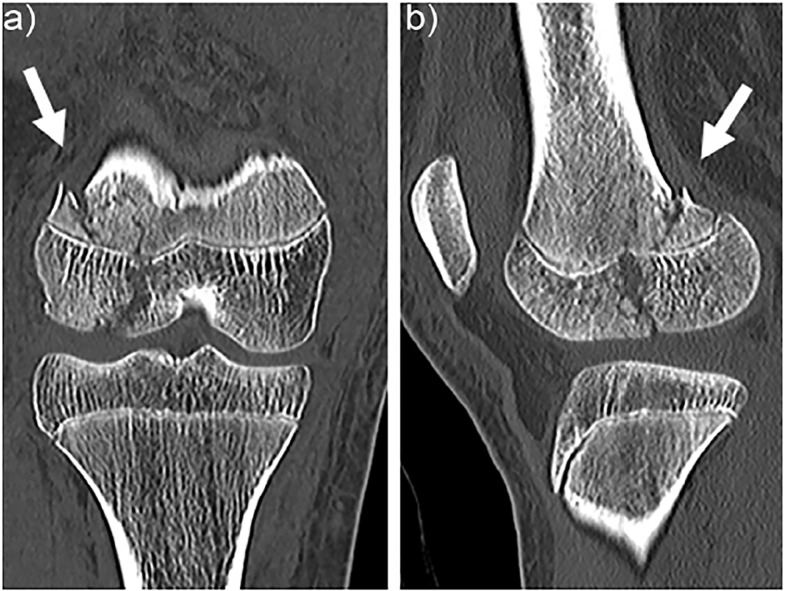
Coronal (A) and sagittal (B) views on computed tomography show injury to the distal femoral epiphyseal growth plate (Salter-Harris type 4 and tenderness at the point near the epiphyseal closing) (arrow).

**Image 3:**
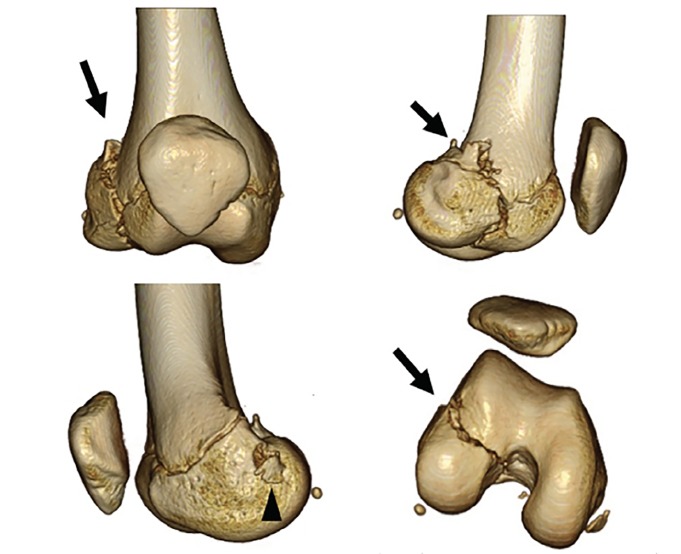
Three-dimensional computed tomography showed that the injured knee had a displaced coronal fracture of the lateral femoral condyle (arrows) and avulsion of the attachment of medial collateral ligament (arrowhead).

A 13-year-old girl presented to the emergency department (ED) after her right knee was forced into valgus after making contact with the opposing goalkeeper while playing soccer. At the scene, she had experienced immediate severe knee pain and was unable to bear weight. Anteroposterior radiographs of the knee revealed a minimally displaced fracture to the lateral femoral condyle (Image 1). Computed tomography (CT) revealed injury of the distal femoral epiphyseal growth plate (Salter-Harris type 4), and the point near the epiphyseal closing was tender in the patient (Image 2). Three-dimensional CTs are useful in delineating the coronal shear component (Image 3). Knee arthroscopy revealed severe complications including posterior cruciate ligament ruptures, medial collateral ligament injury, and longitudinal tear of the lateral meniscus anterior horn, in addition to suspicion of these injuries on preoperative magnetic resonance imaging (MRI). The patient underwent open reduction and internal fixation (ORIF) to achieve anatomic reduction.

## DIAGNOSIS

Coronal fractures of the femoral condyle, first described by Hoffa in 1904, are uncommon clinical entities, typically seen in adults after high-energy trauma.^1^ Historically, poor outcomes have been reported in the literature with non-operative treatment. ORIF has been shown to produce good, long-term clinical results in adults. Hoffa fractures appear to be even more uncommon in a skeletally immature patient. Nevertheless, they should also be treated with ORIF to achieve anatomic reduction, stable internal fixation, and early active mobilization.^2^,^3^ Plain radiographs are not sufficiently sensitive to detect Hoffa fracture fragments, and there is a risk of underestimation. Emergency physicians should not be hesitant to order CTs for determining accurate diagnosis. To confirm ligamentous and cartilaginous injuries, MRI and knee arthroscopy are useful. In the ED, the emergency physician should initially fix the patient’s knee using an above-knee splint, and consult an orthopedic surgeon for subsequent ORIF.

**CPC-EM Capsule**

What do we already know about this clinical entity?

Hoffa fracture is uncommon, and poor outcomes have been reported with conservative treatment. Plain radiographs are not sufficiently sensitive and there is a risk of under diagnosis.

What is the major impact of the image(s)?

The computed tomography (CT) image in this report demonstrates injury of the distal femoral epiphyseal growth plate and a displaced coronal fracture of the lateral femoral condyle, which are underestimated on plain radiographs.

How might this improve emergency medicine practice?

This case report demonstrates emergency physicians should not hesitate to order CT to accurately diagnose Hoffa fracture.

Documented patient informed consent and/or Institutional Review Board approval has been obtained and filed for publication of this case report.
